# Integrin-Mediated TGFβ Activation Modulates the Tumour Microenvironment

**DOI:** 10.3390/cancers11091221

**Published:** 2019-08-21

**Authors:** Nicholas F. Brown, John F. Marshall

**Affiliations:** Centre for Tumour Biology, Barts Cancer Institute, Cancer Research UK Centre of Excellence, Queen Mary University of London, Charterhouse Square, London EC1M 6BQ, UK

**Keywords:** integrins, TGFβ, αvβ6, tumour microenvironment

## Abstract

TGFβ (transforming growth factor-beta) is a pleotropic cytokine with contrasting effects in cancer. In normal tissue and early tumours, TGFβ acts as a tumour suppressor, limiting proliferation and inducing apoptosis. However, these effects are eventually abrogated by the loss or inactivation of downstream signalling within the TGFβ pathway, and in established tumours, TGFβ then acts as a tumour promotor through multiple mechanisms including inducing epithelial-to-mesenchymal transition (EMT), promoting formation of cancer-associated fibroblasts (CAFs) and increasing angiogenesis. TGFβ is secrereted as a large latent complex and is embedded in the extracellular matrix or held on the surface of cells and must be activated before mediating its multiple functions. Thus, whilst TGFβ is abundant in the tumour microenvironment (TME), its functionality is regulated by local activation. The αv-integrins are major activators of latent-TGFβ. The potential benefits of manipulating the immune TME have been highlighted by the clinical success of immune-checkpoint inhibitors in a number of solid tumour types. TGFβ is a potent suppressor of T-cell-mediated immune surveillance and a key cause of resistance to checkpoint inhibitors. Therefore, as certain integrins locally activate TGFβ, they are likely to have a role in the immunosuppressive TME, although this remains to be confirmed. In this review, we discussed the role of TGFβ in cancer, the role of integrins in activating TGFβ in the TME, and the potential benefits of targeting integrins to augment immunotherapies.

## 1. Introduction

The extracellular matrix (ECM) serves both as a scaffold for cells and as an information-rich system that cells decipher through interacting sensory inputs in which integrins and transforming growth factor-beta (TGFβ) are highly influential [1]. Homeostasis regulates the balance of cytokines and cell surface receptors to mediate intricate interactions between cells and the ECM. TGFβ is the most pleiotropic known cytokine and almost every cell produces TGFβ and has receptors for it [2,3]. It is an effective growth inhibitor of epithelial, haematopoietic and immune cells and is locally activated during tissue remodelling to regulate growth and repair. Dysregulation of TGFβ is implicated in a number of pathologies, most notably cancer and tissue fibrosis [3,4]. Whilst almost ubiquitously secreted, TGFβ is held within the ECM in an inactive state, requiring activation to mediate its effects. As discussed below, the αvβ integrins are major activators of TGFβ in both normal tissue and cancer.

**Figure 1:**
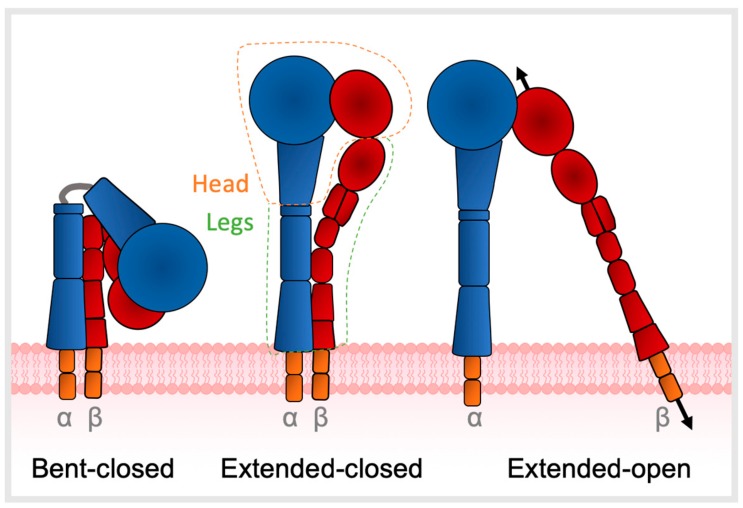
Integrin αvβ6 conformation states. Integrins comprise an α- and β-subunit associated non-covalently at the head, each connected to a flexible leg that traverses the cell membrane to a short cytoplasmic domain. Ligand affinity is mediated by changes in conformation, with affinity highest in the extended-open state and lowest in the bent-closed state. The black arrows indicate the direction of force that integrins mediate between the extracellular matrix and actin cytoskeleton adapted from [9].

Integrins are heterodimers that mediate bidirectional signalling across cell membranes, coupling diverse extracellular ligands to the cytoskeleton [1,5,6]. Integrin ectodomains comprise an α- and β-subunit non-covalently joined at the head, each connected to a flexible leg which traverses the cell membrane to a short cytoplasmic domain (Figure 1) [7]. The endodomains link with cytoskeletal components and thus allow integrins to act as mechanotransducers between the cell and ECM [8,9,10]. Regulation of ligand affinity and signalling is mediated by a series of coupled motions of the headpiece with leg domains that change the overall shape from bent to extended [11]. Integrin structure is described in three main conformations—bent-closed, extended-closed, or extended-open, with low ligand affinity when in the bent state [10,12]. A total of 24 different αβ heterodimers exist, composed of 18 α- and 8 β-subunits, each with different functional and tissue specificity. The primary binding site of integrins to their target ligands is via recognition of short peptide motifs, the most common of which is arginine-glycine-aspartic acid (RGD) motif; eight integrins (αIIβ3, α5β1, α8β1, αvβ1, αvβ3, αvβ5, αvβ6, αvβ8) recognize the RGD motif [13].

Integrin functions are myriad and include cell adhesion, migration, proliferation, differentiation, survival and invasion but the αv-integrins, particularly αvβ6 and αvβ8, are specialised to activate TGFβ [14]. Since integrins and their ligands are amongst the plethora of TGFβ-regulated transcriptional targets, TGFβ-integrin interactions are bilateral.

In this review, we discussed TGFβ activation by integrins and its consequences by promoting cancer through regulating the the immune and non-immune tumour microenvironment (TME).

## 2. TGFβ Structure and Secretion

The transforming growth factor β (TGFβ) family is encoded by 33 genes and includes the three TGFβ isoforms (TGFβ1, TGFβ2, TGFβ3) relevant to this review [15,16,17]. The three isoforms are 75% homologous with similar signalling activities but have variable expression in different cells and tissues, and distinct phenotypes are observed in vivo in knockout models [18]. TGFβ1 is predominantly expressed in endothelial, hematopoietic, and connective-tissue cells, TGFβ2 in epithelial and neuronal cells, and TGFβ3 in mesenchymal cells [19]. TGFβ1 is the most abundant, most widely studied, and most commonly dysregulated isoform in cancer [20,21], and thus, will be the focus of this review.

**Figure 2:**
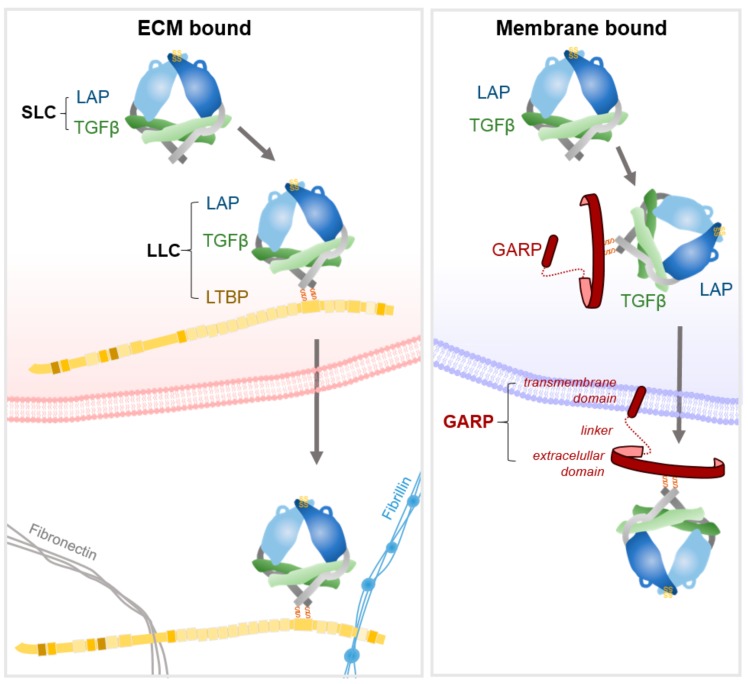
TGFβ (transforming growth factor-beta) structure and secretion. Dimers of TGFβ and latency-associated peptide (LAP) linked by non-covalent bonds form the small latent complex (SLC). Left: Covalent bonds link the small latent complex to latent TGFβ binding protein (LTBP) which together form the large latent complex (LLC). Once secreted, TGFβ is held in an inactive state, anchored by LTBP to fibronectin and fibrillin in the extracellular matrix. Right: In some cells, notably regulatory T-cells, the SLC binds covalently to glycoprotein-A repititions predominant (GARP), and following secretion is bound to the cell membrane. Adapted from [15,16,29]. SS, disulphide bonds.

TGFβ is initially translated as a propeptide comprising the growth factor and latency-associated peptide (LAP). A disulphide-linked dimer of the propeptide is formed, which is cleaved by furin to release the mature TGFβ from LAP. However, the affinity of the propeptide LAP for TGFβ is such that it assembles into a non-covalent complex, termed the small latent complex (SLC) that comprises TGF-β and homodimers of LAP (Figure 2) [2,16,22]. The SLC is then further processed before it exits the cell. In non-leukocyte cells, a pair of disulphide bonds link LAP to a latent-TGFβ binding protein (LTBP) [22]. LTBPs are large glycoproteins that serve as chaperones for pro-TGFβ by enhancing folding and secretion [22]. In leukocytes, the glycoprotein-A repetitions predominant protein (GARP) bind to LAP, again via two disulphide linkages and again serving as chaperone for correct presentation of the complex [23,24,25]. As GARP is a transmembrane molecule, it holds the SLC at the membrane (discussed in [25]). Thus, TGF-β is secreted as a tripartite complex of TGF-β, LAP and either LTBP or GARP, termed the large latent complex (LLC) [9,22,26]. Once secreted, LTBPs localise latent-TGFβ to the ECM by interactions with fibronectin and fibrillin [22,27], the four LTBPs being expressed in a tissue specific fashion [2]. The majority of latent-TGFβ is sequestered within the ECM, with the remaining latent-TGFβ anchored to immune cell surfaces or stored in α-granules within platelets and mast cells [28].

## 3. TGFβ Activation

TGFβ is stored within the ECM at concentrations that are several orders of magnitude higher than required to produce potent biological effects [30]. Thus, most TGFβ regulation occurs at the level of activation of latent-TGFβ sequestered within the ECM by LTBP or on cell-surface scaffold proteins by GARP [9,22,26,30]. Active TGFβ has a considerably shorter half-life than latent TGFβ and is rapidly cleared from the extracellular space if not associated with its receptor [31]. Thus, the activation of latent-TGFβ permits tight spatial and temporal regulation of TGFβ signalling [32]. Latent-TGFβ is activated in vitro by a variety of protease and non-protease-dependent mechanisms, including physiochemical conditions. However, the major activators of TGFβ in vivo are integrins, most prominently αvβ6 and αvβ8.

### 3.1. Knockout Mouse Phenotypes

Early indications of integrin involvement in TGFβ activation came from similarities in knockout mice phenotypes. TGFβ1^−/−^ mice die shortly after birth from multi-organ inflammation and vasculogenesis defects. TGFβ2^−/−^ mice die around the time of birth with multi-organ developmental defects. TGFβ3^−/−^ mice develop cleft palate (reviewed in [32]). Integrin β6^−/−^ mice develop lung and skin inflammation [33]. Moreover, they are protected from bleomycin-induced lung fibrosis, established as TGFβ1 dependent, and gene expression profiles of the β6^−/−^ lungs show the majority of TGFβ responsive genes are not upregulated [14,34]. In addition, β6^+/+^ mice dramatically upregulate αvβ6 in response to bleomycin, suggesting that αvβ6 expression is important for local activation of TGFβ1 in epithelial cells [14]. These correlations between increased TGFβ1 activity and αvβ6 expression were confirmed by showing αvβ6-specific antibodies blocked activation of TGFβ1 [14]. The vital role of the RGD integrin-binding sequence in TGFβ1 activation was demonstrated in vivo by generating mice whose TGFβ1 lacked the integrin recognition motif RGD in LAPβ1 which is replaced with the inactive motif RGE. TGFβ1^RGE/RGE^ mice exhibit the TGFβ1^−/−^ phenotype despite normal levels of latent-TGFβ1 [35,36].

Whilst most integrin-β6^−/−/^β8^−/−^ double knockout mice die in utero, those that survive develop cleft palate [37], a phenotype replicated in the *garp* knockout mouse [38], showing that GARP regulates TGFβ3. Interestingly, replacement of the TGFβ3 gene with TGFβ1 at the TGFβ3 locus partially rescues palate closures, highlighting that TGFβ3- and TGFβ1-LAP share critical features but also display isoform-specific roles [39]. Integrin-β8^−/−^ mice have abnormal cerebral and yolk sac vasculogenesis. Whilst the yolk sac defect is seen in TGFβ1^−/−^ mice, the cerebrovascular defect is not apparent in TGFβ single isoform knockouts, suggesting overlapping functions in the TGFβ isoforms [40]. A key observation was that conditional deletion of αvβ8 in dendritic cells (DCs) resulted in widespread inflammation in the intestines, attributed to failure of DCs to activate TGFβ and thus regulate Treg activity (discussed below) [41]. Furthermore, pharmacological inhibition of αvβ6 in β8^−/−^ mice causes a similar phenotype to TGFβ1^−/−^ mice, consistent with αvβ6 and αvβ8 as dominant latent TGFβ1 activators.

### 3.2. Ligand Affinity

The RGD integrin-binding motif is present on the LAP propeptides of TGFβ1 and TGFβ3, which have been shown to bind αvβ1, αvβ3, αvβ5, αvβ6, αvβ8, and α8β1 [14,36,42,43,44,45,46]. The homologous latency associated peptide from pro-TGFβ2 has SGD (serine-glycine-aspartic acid) in place of RGD and binds to αvβ6, but with a thousand-fold lower affinity than LAPβ1 [47] and thus, is not activated by integrins [48]. LAP-TGFβ1 binds strongly to αvβ6 (10.3 nM) and αvβ8 (13 pM) but with a significantly lower affinity for αvβ3 (8.5 μM). This nanomolar affinity is unusual in integrins, which typically bind with lower affinity to allow the reversal of adhesion in retracting regions of migrating cells. Thus, this high affinity may reflect specialisation to support TGFβ-activation over cell migration [36,47]. The higher affinity of αvβ6 and αvβ8 for LAP-TGFβ1 is due to the ability to bind both to RGD and to a second binding motif, LXXL/I which αvβ3, αIIbβ3, and α5β1 are unable to recognise [47].

### 3.3. Force-Mediated Activation of ECM Bound Latent TGFβ by αvβ6

When integrins bind to the RGD motif on LAP, association with the actin cytoskeleton triggers conformational changes in the LLC that releases TGFβ [49,50]. αvβ6 activates latent-TGFβ even in the presence of a cocktail of protease inhibitors, indicating a non-protease-dependent mechanism [14]. Furthermore, binding alone of integrins to LAP does not lead to TGFβ activation [1,14,15,22,49,50]; traction forces generated through αvβ6 binding to the LAP of latent TGFβ are required. This activation is abolished by actin cytoskeleton inhibitors, truncation of the β6-endodomain residues that bind to the cytoskeleton, or by deletion of the binding site for the latent TGFβ to bind to the ECM that is required to generate tensile force across the pro-domain [22]. Thus, LAP anchored to the ECM by LTBP and secured to the cell surface by integrins is distorted by traction between the matrix and cells that permits liberation of active TGFβ [49,50,51].

**Figure 3:**
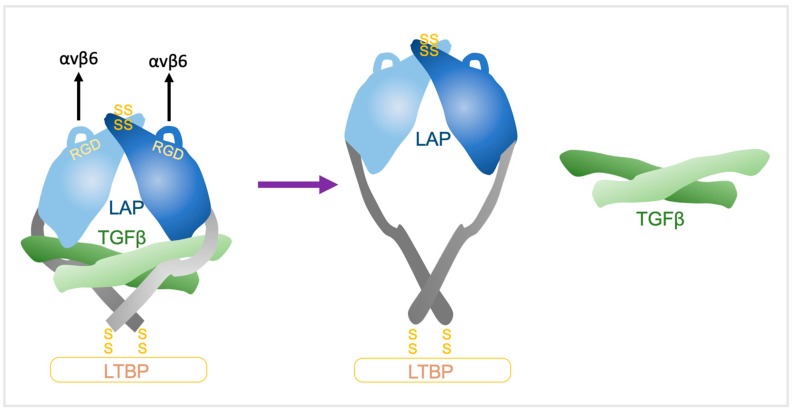
Force-mediated liberation of TGFβ by αvβ6. Left: TGFβ is held in a ‘straitjacket’ by LAP with the ‘latency lasso’ preventing covering TGFβ receptor binding sites. αvβ6 activates TGFβ by binding to RGD motifs on the shoulders of LAP and transducing a force (black arrows) from the actin cytoskeleton. A resistant counter-force is generated by LTBPs which are bound to LAP by disulphide bonds (SS). Right: When tensile force is exerted across the structure, the lasso is elongated, the straitjacket unfastens, and active TGFβ is liberated. Adapted from [15].

The underpinning mechanism was solved by the Springer group whereby crystal structures of latent TGFβ revealed a ring-like shape with two LAP prodomain ‘arms’ connected at the ‘elbows’ to crossed ‘forearms’ formed by two TGFβ monomers and by LAP prodomain ‘straitjacket’ elements that surrounded each TGFβ monomer (Figure 3). The arms come together at the neck, disulphide linked in a ‘bowtie’, with RGD motifs located at each ‘shoulder’. The RGD motifs are accessible for integrins and nearby exposed hydrophobic sidechains on the body of the arm increase affinity for integrins [15]. αvβ6 binds in a 1:2 complex of the αvβ6 head bound to one monomer of the latent-TGFβ dimer [9]. The actin cytoskeleton generates the force necessary for TGFβ release from the αvβ6/pro-TGFβ complex through the β6-subunit cytoplasmic domain, with LTBP anchored to the ECM providing countertraction [9]. Integrin headpiece opening increases affinity for TGFβ by altering a β-leg domain orientation and thence, the direction of the force when traction force is applied to the β-subunit by the actin cytoskeleton [9]. The LAP lasso holds TGFβ within the straitjacket and covers all of the TGFβ receptors contact sites [15]. RGD motifs are located on opposite sides of the lasso, so when tensile forces are exerted across them, the lasso is elongated and TGFβ is liberated [9,52].

This force-dependent mechanism is supported by experiments that demonstrated αvβ5 integrin-dependent release of active TGFβ by contracting myofibroblast cytoskeletons occurs in a wholly mechanical manner in detergent-treated cells that lack cell membranes or cytosol [53]. Moreover, ferromagnetic beads coated with integrins or anti-LAP beads are capable of liberating TGFβ from a cell-free matrix [52].

TGFβ activation by αvβ6 is also dependent on the covalent attachment of LAP to LTBP, specific ECM-binding regions in LTBP-1, and the presence of fibronectin in the matrix [49,51]. LTBP supports αvβ6-mediated activation by concentrating and fixing the latent complex. Recombinant SLC that lacks LTBP fails to activate TGFβ, and LTBP1-derived TGFβ binding cassettes that cannot bind to ECM prevent αvβ6-mediated activation. However, if the complex is artificially fixed to the pericellular environment, activation can occur [49]. Fixation of LTBP to the mechano-resistant ECM creates a resistant ‘holding force’ to the transmembrane ‘pulling force’ of integrins attached to the actin cytoskeleton, and together, these generate sufficient force to unfasten the straitjacket and liberate TGFβ. αvβ6 activation is LTBP1-specific, as αvβ6 can activate TGFβ bound to LTBP1, but not LTBP3 [49].

### 3.4. Activation of GARP-Bound Latent TGFβ on Treg Cells

One of the most powerful pro-tumourigenic functions of TGFβ1 is to promote an immunosuppressive environment in the TME and recent data highlights the central role of αvβ8 in this process. It has been known for some time that TGFβ signalling in T cells was required to maintain immune tolerance, since the genetic abrogation of T-cell TGFβ signalling resulted in lethal autoimmunity [54,55]. The source of the TGFβ required to promote these signals is only recently becoming clearer. It had also been established that regulatory T (Treg) cells can produce activated TGFβ1 that can suppress the activity of helper-T (Th) cells [56,57] and had latent TGFβ1 on their surface membranes [58] in association with GARP [59]. Genetic suppression [59] or antibody [60] inhibition of GARP confirmed that GARP was required for activation of TGFβ1 by Treg cells but was not sufficient to activate TGFβ1. Thus, the expression of GARP in HEK293 cells resulted in the surface expression of latent TGFβ1 but no TGFβ1 activity. Developing upon the mechanisms identified for the activation of ECM bound latent TGFβ all five αv integrins were assessed for their capacity to activate latent TGFβ on the GARP expressing HEK293 cells; only αvβ6 and αvβ8 generated active TGFβ, but only if the GARP retained its transmembrane domain; “soluble” GARP-LAP-TGFβ released from cells expressing a transmembrane-deletion mutant of GARP was not activated by any αv integrins [23]. These data imply (i) that αvβ8 is the principal activator of GARP-LAP-TGFβ complexes on TReg cells since αvβ6 is epithelial-specific, a prediction that is now confirmed since either genetic deletion or antibody inhibition of αvβ8 on Treg cells inhibits activation of cell surface TGFβ [61,62,63], and (ii) that[the GARP-LAP-TGFβ must be tethered to permit activation to occur, similar to that of LTBP bound latent-TGFβ. Intriguingly, while αvβ6 forms strong association with the cytoskeleton through conserved domains [64] within its cytoplasmic tail, αvβ8 has a divergent cytoplasmic tail that lacks the conserved domains [65] that predict cytoskeletal interaction. Thus, if αvβ8 mediates the activation of GARP-LAP-TGFβ through force generation, as αvβ6 does for LTBP1-LAP-TGFβ, the mechanism for generating transmembrane cytoskeletal force remains unclear.

Another similarity between the activation of LTBP1-LAP-TGFβ and GARP-LAP-TGFβ is that the cell responding to the release/exposure of the mature TGFβ needs to be in contact with the cell where TGFβ was activated [14,65]. This suggests that either the mature ‘free’ TGFβ1 cytokine is not released from the Treg surface and remains associated with the GARP-LAP complex, or that, if it is released, it does not reach the threshold concentration required to suppress Th cells; biologically the former model is more conservative and provides a much more secure spatio-temporal mechanism for regulating TGFβ activation in our tissues. The relative importance of one or both mechanisms in vivo remains unclear.

GARP is expressed by cells other than Tregs, including platelets, megakaryocytes, fibroblasts, heapatic stellate cells, endothelial cells, some carcinomas, as described in the excellent review by Stockis et al. [25]. Few formal studies have examined if they also exhibit integrin-dependent local activation of TGFβ. However, in the context of cancer it is perhaps worth noting the platelets. In a recent study of mice whereby the GARP protein was selectively deleted from platelets, the amount of circulating active TGFβ1 reduced significantly and the tumour growth rate was reduced, corresponding with an increased immune infiltrate into the TME [66]. Thus, platelets require GARP to produce activated TGFβ and appear to be an important source of both systemic and tumour-associated TGFβ1 mediating immunosuppression. What is not clear is what, if any, roles integrins play in activating platelet TGFβ1.

### 3.5. Metalloprotease-Dependent TGFβ Activation

αvβ8 binds LAP-β1 with high affinity (Kd 13 pM). αvβ8 mediated TGFβ-activation is illustrated by the inhibition of activation with anti-αvβ8 antibodies [36]. However, the mechanism of activating ECM-bound TGFβ is distinct from that of αvβ6. As mentioned previously, the β8 cytoplasmic-domain is dissimilar to any other β-integrins [65] and may be incapable of linking to the actin cytoskeleton [67], and thus, incapable, directly, of TGFβ-activation by mechanical transduction [36].

The current understanding of αvβ8-mediated TGFβ-activation is that LAPβ1 and LAPβ3 bind with high affinity to αvβ8 on cell surfaces, and αvβ8 brings the latent complexes into juxtaposition with a membrane metalloprotease (MMP) which cleaves LAP, releasing TGFβ. This is based on a number of findings from a single study. Consistent with a proteolytic event, active TGFβ is liberated into tumour cell line supernatants and into the aqueous phase of lung cancer xenografts by an αvβ8-dependent mechanism. αvβ8 and MMP-14 co-localise in LAPβ1 substrate contacts and β8-specific RGD inhibitors and MMP-14 inhibitors both block αvβ8-mediated TGFβ activation, indicating that β8 and MMP-14 are both required. MMP-14 deficient H1264 lung cancer cell lines are unable to activate TGFβ via αvβ8, whilst the restitution of MMP-14 rescues αvβ8-mediated TGFβ activation. Consistent with LAP-β1 as the proteolytic substrate of αvβ8-MMP-14-mediated TGFβ activation, β8-overexpressing/MMP-14 expressing H1264 cells cleave and inactivate LAP-β1, whereas β8-overexpressing/MMP-14 deficient H1264 do not [36].

The mechanism was further supported by the finding that the ability of monocytes to activate TGFβ also relates to both αvβ8 and MMP-14. TGFβ activation in monocytes is almost abrogated with an anti-β8 antibody. Whilst all monocyte subsets express similar amounts αvβ8, only CD14+ monocytes activate TGFβ, suggesting that as with αvβ6, the mere expression of αvβ8 is insufficient to activate TGFβ. MMP-14 expression varies ten-fold between CD14+ and CD14− monocytes, and expression correlates with the ability to activate TGFβ. Antibody inhibition of MMP-14 diminished TGFβ to a similar degree to anti-β8 antibodies [68].

However, MMP knockout mice do not recapitulate the TGFβ^−/−^ phenotype. Furthermore, whilst it has been shown that αvβ8 expression on Tregs is vital for TGFβ activation, the authors stated that the role of MMP-14 was unclear given that Tregs did not have increased expression of MMP-14 compared with naïve T cells [62]. Moreover, protease inhibitors do not reduce TGFβ activation [63], indicating that at least in Tregs, αvβ8-mediated TGFβ activation can occur in a non-MMP dependent manner.

### 3.6. Other Mediators of TGFβ Activation

The higher affinity for LAPβ1 of αvβ6 and αvβ8 possibly explains their role as the major activators of TGFβ. However, TGFβ activation can be activated by a number of other integrins.

αvβ5 and αvβ3 are widely expressed and although their respective knockout mice do not display phenotypes associated with decreased TGFβ activation, they have been shown to activate TGFβ in vitro when expressed in fibroblasts [45,69]. While the mechanisms were uncertain, there are hints that MMPs may play a role for some integrins. There is a correlation between activated αvβ3 and MMP-9 expression in breast cancer cells [70] and the localisation of MMP-2 to the surface of melanoma cells upon interaction with αvβ3 [71]. Both MMP-2 and MMP-9 are capable of activating TGFβ in vitro [72]. Evidence for mechanical release of TGFβ by αvβ5, αvβ3, and an unidentified β1 integrin was demonstrated in contracting myofibroblast cytoskeletons, with stretching of adherent myofibroblasts increasing their ability to liberate TGFβ [53]. Based on separate studies, it is likely that the β1 integrin is αvβ1. Mice with hepatic stellate cells with integrin-αv^−/−^ do not develop carbon tetrachloride-induced liver fibrosis. However, mice with global knockout of β3, β5 or β6 or conditional knockout in hepatic stellate cells of β8 do develop liver fibrosis, unless pharmacological blockade of αv-integrins is administered [73]. αvβ1 has also been implicated as a promotor of bleomycin-induced lung fibrosis. Given that this pathology is also established as αvβ6-mediated, this suggests that multiple integrins can operate simultaneously to regulate TGFβ-mediated stromal responses [14,73].

αvβ3, αvβ5, αvβ6, several β1 integrins and MMP genes are themselves TGFβ transcriptional targets. Thus, integrin-mediated TGFβ activation can generate self-amplifying feed-forward loops. In addition, activated TGFβ induces local fibroblasts to differentiate into myofibroblasts which contracts the ECM. This places anchored LLC under tension and lowers the threshold for activating latent-TGFβ, augmenting the feed-forward loop and permitting activation by integrins with lower LAP-binding affinity [45]. Crosstalk amongst integrins also modulates TGFβ activation. Integrin-β1^−/−^ mice or pharmacological inhibition of β1 induces compensatory upregulation of β3 and increases TGFβ activation in breast cancer cells. However, the overexpression of β3 did not replicate this phenotype after β1 loss, implying that there are other modulators of TGFβ in this setting [74].

Physiochemical conditions such as heat and low pH can also unfasten the straitjacket and either activate TGFβ or lower the activation threshold [32]. Of note is the diverse range of non-integrin proteins that have been associated with TGFβ activation and are discussed comprehensively elsewhere [22].

## 4. TGFβ Signalling

Having liberated/exposed the mature TGFβ cytokine, it can interact with its cognate receptors and promote a variety of intracellular pathways. TGFβ signaling is described by many excellent reviews including [3,4,17,21] and the following is just a brief overview.

Once activated, TGFβ triggers signalling in cells by binding to the TGFβ serine/threonine kinase receptor complex. Upon binding to TGFβ receptor 2 (TGFβR2), TGFβ receptor 1 (TGFβR1) is recruited, transphosphorylated and activated. Serine/threonine protein kinases in the intracellular domain leads to the recruitment and phosphorylation of the intracellular downstream mediators Smad2 and Smad3. Oligomerisation of phosphorylated Smad2 and Smad3 with Smad4 permits translocation to the nucleus where they act as a transcriptional complex. Inhibitory Smads, including Smad6 and Smad7, regulate the pathway [16,17,21,32,75,76,77,78,79]. The oligomers of Smads acquire different stoichiometries, permitting different signalling thresholds of gene expression [21,80]. TGFβR3 (β-glycan) can amplify the signalling cascade by binding TGF-β and promoting presentation to TGFβR2 [16,17,78]. TGFβ2 has a lower binding affinity than TGFβ1 and TGFβ3 and therefore, relies on β-glycan for high-affinity binding to the TGFβ receptor complex. Therefore, in endothelial and haematopoietic cells that do not express β-glycan, TGFβ2 has a limited activity [81,82].

Smad target genes interact with a range of transcription factors. Interactions between these co-activators and co-repressors define the degree of transcription. Although Smads bind to DNA with a 100-fold lower affinity than high-affinity transcription factors, they are required for transcriptional activation [21]. TGFβ receptors remain active for several hours after ligand binding, and repeated receptor activation maintains Smad complexes within the nucleus [83].

The canonical TGFβ pathway is Smad-mediated. Smad-independent TGFβ-activated signalling pathways mediate a non-canonical pathway that includes the PI3K-AKT, Ras/ERK, p38 kinase, and small GTPase (RHOA, PKN, Rock) pathways [16,21,75,84]. Both Smad- and non-Smad dependent pathways may induce TGFβ1 expression, thus amplifying the TGFβ response [85]. Although the converging pathways usually results in cooperativity, pathways may counteract each other [21].

## 5. TGFβ in Cancer

TGFβ has a dichotomous role in cancer, emerging as a positive prognostic factor in early tumours, yet as a poor prognostic marker in advanced tumours [77]. A definitive “switch” from tumour suppressor to promoter is not evident. Rather, an accumulation of genetic, epigenetic and cellular events in canonical and non-canonical pathways within cancer cells and the TME drive a phenotype of decreased TGFβ responsiveness and the increased expression or activation of TGFβ ligands [17,86,87]. The role of TGFβ in cancers can broadly be categorised into effects on cell proliferation, induction of epithelial to mesenchymal transition (EMT), modulation of the TME, and dampening of immune surveillance [88,89,90]. Below we outline these actions, focusing on the role of integrins.

### 5.1. Proliferation

In normal tissues, TGFβ counteracts proliferation primarily by inhibiting cell-cycle progression with G1 arrest and stimulation of cyclin-dependent kinase inhibitors [2,20]. This is demonstrated in vivo where integrin β8-expressing cancer cells are less tumorigenic and have slower growth mediated by increased TGFβ activation [36]. However in cancer, growth-inhibitory effects of TGFβ are eventually overcome by either “decapitation” of core pathway components—TGFβ receptors or Smad transcription factors, or the loss of downstream signalling targets [77]. Within the TGFβ pathway, loss of function mutations are most common, particularly in gastrointestinal cancers [91], notably in pancreatic cancers and colorectal cancers (up to 100% and 83% of patients respectively) [92,93]. Truncated, functionally inactive TGFβR2 is associated with high microsatellite instability cancers [2,77] and Smad4 is mutated in about half of pancreatic, colon, and oesophageal cancers [77].

A number of experimental findings support the increased tumorigenicity associated with abrogated or dysfunctional TGFβ pathways. In normal keratinocytes, TGFβ1 knockout or expression of dominant-negative TGFβR2 causes genomic instability and malignant transformation [94]. Cancer cells that have lost sensitivity to TGFβ due to mutations in TGFβR1 but can be re-sensitised by re-expression of TGFβR1, also resulting in reduced tumour formation [95].

Integrins are also implicated. A large fraction of integrin-β6^−/−^ mice have spontaneously occurring tumours, with the phenotype linked to deficient TGFβ activity [96]. αvβ6 blockade increases tumour cell proliferation in both early and late stage disease, depending on the presence of Smad4 [97]. In addition, inappropriate α6β4 expression in stratified squamous epithelia has been found to inhibit TGF-β signalling and increase tumorgenicity by impeding TGFβ-mediated suppression of clonal expansion of initiated cells within the epidermal basal layer [98,99].

### 5.2. EMT & Metastasis

Epithelial-mesenchymal transition (EMT) is a process by which epithelial cells lose or reduce cell–cell and cell–substrate adhesion and transition to a more mesenchymal-like phenotype, aiding migration, invasion, and metastatic spread [100]. The loss of E-cadherin, typically located at cell–cell adhesion junctions and essential for maintaining an epithelial phenotype, is a hallmark of EMT [101]. The TGFβ pathway can mediate the entire switch from epithelial to mesenchymal phenotype by repression of epithelial gene signatures, including E-cadherin, and the elevation of mesenchymal genes such as α-SMA and vimentin [42,102]. Smad-mediated pathways induce the loss of cellular adhesion and non-canonical pathways, particularly as RHO and AKT promote migration and invasion [103,104,105].

Integrins potentiate TGFβ-mediated E-cadherin downregulation. In renal cancer cells, cyclo-RGD peptide αvβ3 ligand and TGFβ1 inhibit E-cadherin with a synergistic effect [106]. During EMT of mammary epithelial cells, β1-integrins induce TGFβ dependent p38 MAPK activity [107]. In basal cell carcinoma organotypics, αvβ6-mediated TGFβ activation induces differentiation of fibroblast into myofibroblasts with subsequent induction of tumour cell invasion [108]. Similar effects have been shown with α5β1, αvβ3, and αvβ5. These other integrins are not normally highly expressed in epithelial cells but are induced by TGFβ signalling, consistent with the feed-forward loop discussed earlier. Antagonising TGFβ in these cells blocks induction of these integrins and reduces invasion and metastasis [109], indicating that the inhibition of integrin-mediated TGFβ pathways may reduce metastasis. Indeed, TGFβ responsive stromal signalling has been demonstrated to drive metastasis. In breast cancer cells, it has been shown to promote single cell motility and intravasation and to be essential for blood-borne metastases [110]. In Her2-positive breast cancer xenografts, αvβ6-expression is associated with increased metastases, and treatment with the anti-αvβ6/αvβ8 antibody causes decreased metastases and pSmad2 [111]. In breast ductal carcinoma in situ, αvβ6 expression is associated with progression to invasive cancer. In studies to elucidate the mechanism, overexpression of β6 in myoepithelial cells was found to activate TGFβ1 and upregulate MMP9. The blockade of either MMP or TGFβ inhibited the ability of β6+ myoepithelial cells to promote invasion, suggesting that αvβ6 mediates TGFβ promotion of an invasive phenotype [112].

In colon cancer organoids and in vivo models, transcriptional activation of integrin-αvβ6 enhances tumorgenicity, with autocrine TGFβ implicated as the mediator. In patients with colon cancer, tumour αvβ6-expression is a powerful prognostic marker with a median survival of 16.5 vs 4.8 years in those with low vs high expression [113]. Similarly, in squamous cell carcinoma organotypics, collagen-7 deletion induced increased invasion, αvβ6 upregulation, and increased matrix fibronectin. These findings were suppressed by co-inhibition with TGFβR1 inhibitors. In vivo similar results were observed, along with increased pSmad2/3 signalling in the stroma of collagen-7 deficient SCC tumours, implying that collagen-7 acts as a TGFβ suppressor, and consistent with αvβ6-dependent TGFβ1 activation inducing fibroblast to myofibroblast transition [114].

TGFβ signalling is central to the formation of bone metastases and osteolytic destruction of adjacent bone in both prostate and breast cancers, with TGFβ blockade inhibiting bone metasteses development [115,116,117]. αvβ6 has been shown to be expressed in prostate cancer bone metastases, and in vivo models of bone metastasis have shown that αvβ6 promotes osteolysis through upregulation of MMP-2 and parathyroid hormone-related protein [118]. A subsequent study revealed that αvβ6 was required for TGFβ1-mediated MMP-2 expression in prostate cancer cells [119]. Of note is the fact that in this study, αvβ6 induced TGFβ signalling by interacting with TGFβR2; although a direct association between and αvβ6-TGFβR2 was not fully confirmed [119].

### 5.3. Angiogenesis

Blood vessel proliferation permits tumour growth by providing adequate nutrient supplies, removal of waste products, and routes for metastasis [120]. Pancreatic cancer cells with activated TGFβ induce angiogenesis when implanted into mice [121] whilst TGFβ-blocking antibodies or targeted deletion of TGFβ in mice results in decreased angiogenesis [122]. Epithelial cells with activated TGFβ show increased gene expression of the pro-angiogenic vascular endothelial growth factor (VEGF) and thrombospondin [17]. Of note, the TGFβ effects appear concentration-dependent. In endothelial cells, sheets derived from mouse metatarsals low TGFβ concentrations are pro-angiogenic, but high concentrations are anti-angiogenic. Furthermore, in this setting, integrin-α5β1 is a key mediator of TGFβR1 and VEGF promotion of angiogenesis. TGFβR1 inhibitors and VEGF synergistically upregulate α5 and β3 integrin gene expression, with downregulation or antibody blockade of α5 inhibiting this co-operative effect [123,124]. Similarly, αvβ3 and αvβ5 deletion accelerates tumour angiogenesis through increased expression of VEGF2 and sensitivity to VEGF-A [125,126].

αvβ8 is also implicated in tumour angiogenesis. Integrin-β8^−/−^ mice develop abnormal cerebral vasculogenesis [37,127]. In glioma, orthotopically implanted β8-high astrocytoma cells develop microscopic non-haemorrhagic tumours with uniform vessels, whereas β8-low cells cause large haemorrhagic tumours with an abundant vasculature and a lower Smad2/3 expression in endothelial cells, implying TGFβ-mediation [128].

### 5.4. Stroma

TGFβ modulates the ECM by different mechanisms. TGFβ promotes fibroblast-to-myofibroblast transdifferentiation and stimulates cancer-associated fibroblasts (CAF) to produce ECM- and cell-adhesion proteins including integrins, collagen, and fibronectin [17,129]. Integrins are also implicated as mediating this process in CAFs—another feed-forward loop [10,130]. TGFβ-induced mesangial cell collagen expression has been shown to require integrin-mediated FAK activation [131] and α3β1 potentiates TGFβ-induction of MMP-9 in immortalised keratinocytes [132].

Stromal TGFβR2 expression decreases with tumour progression and is a poor prognostic marker in colorectal cancer [17,133]. The attenuation of TGFβ at either receptor or Smad level results in increased myeloid-derived suppressor cells and stromal fibroblast activation, driven by increases in CXCL1 and CXCL5 expression, which are normally inhibited by TGFβ. This is demonstrated in vivo by increased desmoplasia and angiogenesis in mice pancreatic epithelium expressing a dominant-negative TGFβ-receptor [17].

A number of studies have implicated αvβ6 as a mediator of TGFβ-induced stromal changes. αvβ6 blockade with the antibody 6.3G9 in Detroit-562 pharyngeal tumour cells had no effect on TGFβ-mediated proliferation in vitro, but did inhibit in vivo xenograft tumour growth, suggesting that the TME has an important regulatory role. αvβ6 expression in the stroma correlated with α-SMA, although treatment had no effect on stromal collagen and smooth muscle actin levels [134]. A similar study in Detroit-562 also found limited effects in vitro when treated with 264RAD and a dose-dependent reduction of tumour growth in vivo, which, in this study, was associated with reduced stromal fibronectin and α-SMA [134,135,136]. This difference may be due to the additional blockade of αvβ8 with 264RAD, which was not explored at the time. 264RAD also reduces stromal α-SMA expression in MCF7 and HER2-18 breast cancer xenografts with associated Smad2 reductions [111]. Similarly, a subset of non-small cell lung cancer (NSCLC) cell lines co-cultured with fibroblasts induced an activated CAF phenotype with α-SMA expression. CAF activation was associated with αvβ6, and activation was blocked by either 264RAD or the TGFβR kinase inhibitor SB321542. However, after three days co-culture, only SB321542 was effective, indicating that whilst initial CAF activation was αvβ6-dependent, once activated, alternative TGFβ activating pathways maintain the phenotype [53,136]. Treatment of αvβ6-expressing transgenic pancreatic ductal adenocarcinoma (PDAC) with 264RAD plus gemcitabine significantly increased overall survival compared with gemcitabine alone and was associated with reductions in both pSmad3 and nuclear Smad4 levels, as well as reductions in αSMA-positive fibroblasts and blood vessel density, both targets of TGFβ signalling [137].

Whilst the consensus is that TGFβ1 activated CAF rich stroma is tumour-promoting, conflicting data exist. For example, in transgenic PDAC mice models, depletion of α-SMA-expressing cells, designed to eliminate CAFs, unexpectedly induced invasive undifferentiated tumours with poorer survival, despite associated reductions in fibrosis in both advanced and precursor lesions. Fewer tumour myofibroblasts were also associated with poorer survival in patients with pancreatic cancer. In the mice, the reduced fibrosis was strongly associated with increased Tregs [138]. Separate studies have also found that sonic hedgehog pathway inhibition in mouse pancreatic cancer models reduces stromal density but causes more aggressive tumours [139]. A subsequent study showed that PDAC stroma has at least two types of CAFs, inflammatory (iCAF) and αSMA-expressing myofibroblasts (myCAFs) that had distinct transcriptional and secretory profiles and were likely to affect tumour growth differently [140]. Thus, the earlier study [138] would have eliminated only the myCAFs and left the iCAFs present. In a recent study, at least four types of pancreatic stellate cell (a specialized pancreatic fibroblast) existed, suggesting even greater heterogeneity [141]. It has not been established whether each subtype responds differently to TGFβ, nor which are tumour-promoting or suppressive. Thus, caution would be prudent when therapeutically targeting CAFs in PDAC, and one assumes cancers in other tissues as well, as it is not clear which types of CAF are the tumour-promoting cells.

Recently, studies have identified exosomes as mediators of TGFβ activation between cancer cells and the TME through horizontal propagation of integrin-assoicated phenotypes in a paracrine manner. Integrin-expressing exosomes can directly transfer integrins to target cells [142,143], with transfer from cancer cells to benign cells inducing an αv integrin-mediated aggressive migratory phenotype [144]. Exosomally transported αvβ6 from gut epithelial cells have been shown to be transferred to mucosal dendritic cells, expressed on the surface, activate local TGFβ, and confer tolerogenic properties to dendritic cells including induction of TGFβ production by Tregs [145]. Within cancer, prostate cancer cells have been shown to efficiently transfer αvβ6 via exosomes to αvβ6 negative cells and localize to the cell surface with subsequent enhanced adhesion and LAP-TGFβ migration in vitro [146], although it remains to be seen whether this occurs in stromal cells within the TME. Cancer exosomes are also implicated in increased TGFβ signaling. Exosomes purified from stromal fibroblasts from patients with oral squamous cell cancer (SCC) have been demonstrated to contain TGFβR2, and when these exosomes are transferred into TGFβR2-deficient SCC keratinocytes, TGFβ signaling increases [147].

### 5.5. Immunomodulation

Tumour progression is aided by promoting regulatory immune cell subsets and dampening anti-tumour immunity. TGFβ influences these processes in both the innate and adaptive immune systems. High tumour TGFβ concentrations attracts myeloid and lymphoid cells as described previously [17,87,133,148]. Accordingly, it may be that the immunosuppressive function of TGFβ can be modulated or even abrogated by modulating the extent of TGFβ activation rather than TGFβ production [149], inferring a possible role for integrins.

Many of the effects of TGFβ on immune cells result from an ability to polarise cells towards an alternative differentiation status that is immunosuppressive and pro-tumorgenic (excellently reviewed in [150]). TGFβ induces pro-tumourigenic ‘M2’-macrophages and their presence is a poor prognostic marker in many cancers including ovarian, breast, gall-bladder, oral, oesophageal, and non-small cell lung carcinoma [151,152]. αvβ6 is independently associated with the induction of M2 macrophages, with demonstration in prostate cancer that αvβ6 containing exosomes are transferred to monocytes and promote M2 polorisation, whereas αvβ6 downregulation in exosomes inhibited M2 polarisation [153]. The study implicates αvβ6-mediated modulation of the STAT1/MX1/2 signaling pathway, but whether αvβ6-mediated TGFβ activation contributes is unclear. Neutrophils can also acquire a tumour permissive ‘N2’ phenotype in a TGFβ-dependent manner, with a subset of low-density neutrophils shown to accumulate with tumour progression [154,155]. TGFβ also inhibits NK cell maturation [156,157].

The loss of Smad4 signalling in T cells, but not in epithelial cells, is associated with spontaneous epithelial cancer formation throughout the gastrointestinal tract in mice [158]. TGFβ has been shown to directly suppress CD8+ cytotoxic T-lymphocyte (CTL) function through transcriptional repression of key proteins including perforin, granzyme, and cytototoxins [159]. Tregs act to counter CTL activity and maintain immunological tolerance. Treg accumulation is associated with poor survival, particularly in cervical, renal, melanoma, breast, liver, and gastric cancer [160]. Thus, TGFβ promotion of Treg activity is a significant contributor to tumour progression. Similar multi-organ inflammation of TGFβ^−/−^ mice is seen with TGFβ signalling abrogation specifically in T-cells, implying T-cell mediation of the TGFβ^−/−^ phenotype [54,161]. Treatment of naïve T-cells with TGFβ can induce differentiation to Tregs [87,159,162], which, in liver cancer has been shown to be mediated by CCL22 promotion [163].

αvβ8-mediated TGFβ activation is crucial in preventing anti-tumour immunity through enhanced Treg activity. Integrin-αvβ8 is expressed in Tregs, but not naïve T-cells. Studies have indicated that Tregs require αvβ8 to liberate TGFβ1 from the GARP complex, that αvβ8-deficient Tregs are incapable of inducing differentiation of naïve T cells, and that β8 expressed by Tregs can suppress aberrant T-cell-mediated inflammation [61,62]. Accordingly, mice with conditional knockout of αvβ8 on antigen-presenting DCs develop increased T-cell activation with consequential autoimmunity [41,68,164]. It is possible that tolerance is induced by αvβ8 on DCs releasing TGFβ from Tregs via cell-cell contact, inducing a larger Treg pool [23]. As described above, Tregs have been shown to be specialised activators of TGFβ via αvβ8 expression. Tregs express 50–100-fold higher levels of integrin-β8 and increased ability to activate latent-TGFβ than naïve and effector memory T cells. Unlike Tregs, β8-knockout Tregs are unable to supress CD4+ T cell expansion in vivo, indicating αvβ8 is necessary to limit this effector T-cell function [62]. In addition, anti-β8 antibodies have been shown to block Treg-mediated immunosuppression in vivo [63].

Macrophages also activate TGFβ through an αvβ8-dependent mechanism that can counter pro-inflammatory cytokine production. This is supported by the finding that αvβ8 is upregulated on M2-macrophages and downregulated on pro-inflammatory M1-macrophages, and also by the ability to almost fully suppress TGFβ activation in monocytes treated with anti-αvβ8 antibodies [68].

Crucially, TGFβ inhibition has been shown to modulate antitumour immune responses with enough potency to mediate tumour regression. Preclinically, transgenic mice with EL-4 thymoma or B16-F10 melanoma xenografts grown in mice with T cells deficient in TGFβ signalling generate tumour-specific CTLs that eradicate tumours [165]. In colorectal cancer mouse models, TGFβ is a primary mechanism of immune evasion that promotes T-cell exclusion and inhibits CTL maturation, and although PD-1 or PD-L1 immune checkpoint inhibition has limited efficacy, TGFβR1 inhibition with galunisertib produces potent and enduring anti-tumour T-cell responses, and rendered tumours susceptible to anti-PD-L1 therapy. Galunisertib efficacy was abolished with the depletion of CD8 T-cells, indicating efficacy was through augmentation of anti-tumour immunity rather than other TGFβ mechanisms [166]. Similarly, in a melanoma model, B16 tumours-derived Tregs were able to suppress CTL-mediated killing of explanted tumour cells, with restoration by neutralising antibodies against TGFβ on the Tregs [167].

Excitingly, TGFβ has recently been identified as a key source of resistance to immune checkpoint inhibitors in patients. In a large trial of patients with metastatic urothelial cancer treated with the anti-PD-L1 antibody atezolizumab, a lack of response was associated with a TGFβ signature on fibroblasts in the TME. Tumours were found to exhibit three distinct immune phenotypes—inflamed, excluded, or desert [168,169]. The TGFβ signature was only associated with lack of response in the excluded tumours, consistent with TGFβ-mediated fibroblast activation in the TME. In associated studies in mice with EMT6 breast cancer tumours, which display an immune-excluded phenotype, treatment with either TGFβ or PD-L1 blocking therapy had a minimal effect, but combination treatment led to increased tumour infiltrating T-cells and tumour regression. Combination therapy had limited impact on T-cell or macrophage TGFβ signatures, and thus, the efficacy was attributed to reprogramming stromal fibroblasts and increased TME CTLs [170].

## 6. Therapeutic Targeting of TGFβ

TGFβ inhibitors have as yet failed to deliver the anticipated clinical efficacy [86,171,172]. Recent findings have highlighted that the principal anti-tumour effect of global TGFβ inhibitors may not be against cancer cells as originally believed, but by modulating the TME, as discussed above. Thus, combination therapy with agents that target cell proliferation may elucidate a clearer role for TGFβ inhibitors. Given the role of αv-integrins in liberating active TGFβ, therapeutic inhibition of surface αv-integrin expression may be a more effective method of suppressing local TGFβ activation than current therapies which predominantly inhibit TGFβ receptor activation or downstream signal transduction. Furthermore, the inhibition of αv-integrins may interrupt the feedforward loop of TGFβ activation described several times in this review and thus, prevent self-amplifying tumour-stroma interactions that lead to malignant progression. The most significant potential role will be in patients with tumours where αvβ6 is a poor prognostic marker, notably colon [113], cervix [173], lung [124], and breast cancers [111], and in cancers with high αvβ6 expression, notably pancreatic [174], oesophageal [134], and skin cancer [134]. Certainly, some pre-clinical data suggest that blockade of αvβ6 does suppress local TGFβ signaling [114,132,135].

Of the integrins that activate TGFβ, only anti-αvβ3 antibodies and peptides have been evaluated in patients, none with clinical success [175,176]. The pan-αv blocking antibody abituzumab was evaluated with or without standard therapy in metastatic colorectal cancer. Whilst overall, there was no survival benefit, in patients with high αvβ6 tumour expression, the risk of death was reduced by 59% with abituzumab [177]. However, no endpoints relating to TGFβ are available. Cilengitide was evaluated in glioblastoma in two large clinical trials: CENTRIC (Phase 3) [178], and CORE (Phase 2) [179]. Tumour avβ3 expression was associated with improved survival in patients treated with cilengitide in CORE, but not CENTRIC. However, in CORE there was no evidence that pSMAD levels changed in relation to αvβ3/αvβ5 levels, indicating that survival may not be controlled by αvβ3/αvβ5-dependent TGFβ process [180]. It is worth noting that the discovery that GARP is required for Treg activation of TGFβ and thus functionality and that antibodies to GARP can inhibit this process has resulted in investigations of GARP as a therapeutic target for suppressing Treg-dependent pathologies (discussed in [25]).

Anti-PD-1, PD-L1, and CTLA-4 immune checkpoint inhibitors are licensed in a number of cancers, with remarkably potent and durable anti-tumour effects in a subset of patients [181]. Given that around 70–80% of patients experience side effects, yet less than a third of patients benefit from the expensive therapy, elucidating biomarkers for response or resistance is vital [182,183,184,185]. Thus, the ability to use a TGFβ signature to identify patients with urothelial cancer that are unlikely to benefit from PD-L1 inhibition is clinically very useful, and it will be interesting to see if it translates to other cancers. The finding that combination TGFβ and PD-L1 overcomes resistance to PD-L1 checkpoint-blockade is exciting and will undoubtedly be evaluated clinically to see if the benefits of checkpoint inhibitors can be extended to more patients [170]. However, over 70% of patients treated with checkpoint inhibitors develop autoimmune related toxicity [186], and given the TGFβ^−/−^ inflammatory phenotype discussed earlier, a potential concern is that combination therapy may be too toxic. Again, local targeting of TGFβ by integrin inhibition may overcome this issue, although as yet, very little has been published on the effect on the immune microenvironment when integrins are targeted in cancer. In advanced carcinomas, targeting of both αvβ6 and αvβ8 to eliminate the TGFβ producing capacity of the tumour, monocytes, dendritic cells and Tregs, would seem an appropriate future strategy.

## 7. Conclusions

TGFβ has a dichotomous role in cancer. In normal tissue and early tumours, it acts as a potent tumour suppressor, but with loss or inactivation of TGFβ downstream signalling it becomes a tumour promotor, inducing EMT, metastasis, angiogenesis, stromal changes and an immunosuppressive TME. TGFβ has been implicated as a key mediator of resistance to immune checkpoint inhibitors in patients, and combination therapy in mice with TGFβ inhibitors has been shown to overcome this resistance. However, global inhibitors of TGFβ have failed to achieve the clinical benefits expected owing to co-suppression of TGFβ-dependent homeostatic activities that resulted in off-target toxicity. Thus local tissue inhibition of TGFβ activation may offer improved therapeutic outcomes.

Integrins αvβ6 and αvβ8 are specialised to liberate TGFβ from the latent complex in which it is embedded in the ECM or on the surface of immunosuppressive Tregs and monocytes. Experimentally studies inhibiting of αvβ6 or αvβ8 have resulted in reduced TGFβ signaling, reduced cancer growth, reduced tumour immunosuppression and changes in the TME that suggest a less permissive environment. These data offer hope that similar observations might be possible in humans. Additionally, antibody inhibition of GARP may offer a more selective means to reduce tumour immunosuppression mediated by Tregs and monocytes. Integrins other than αvβ6 and αvβ8 may activate TGFβ when the threshold for activation is lowered, such as when the ECM is under tension, as it would be during a fibrotic stage of growth. This can result in cross-talk between integrins and TGFβ production and activation that can mediate a self-amplifying feed-forward loop of TGFβ activation.

As yet, it remains unclear how anti-integrin therapies affect the tumour immune microenvironment. However, it is worth investigating if such therapies may be superior to specific TGFβ or TGFβ pathway inhibitors in reducing TME immunosuppression and augmenting the anti-tumour immunity mediated by checkpoint inhibitors and other biotherapeutics such as chimeric antigen receptor-T cells.

## References

[1] Hynes R.O. (2002). Integrins: Bidirectional, allosteric signaling machines. Cell.

[2] Blobe G.C., Schiemann W.P., Lodish H.F. (2000). Role of transforming growth factor beta in human disease. N. Engl. J. Med..

[3] Inman G.J. (2011). Switching TGFbeta from a tumor suppressor to a tumor promoter. Curr. Opin. Genet. Dev..

[4] Massague J. (2000). How cells read TGF-beta signals. Nat. Rev. Mol. Cell Biol..

[5] Calderwood D.A. (2004). Integrin activation. J. Cell Sci..

[6] Zhu J., Zhu J., Springer T.A. (2013). Complete integrin headpiece opening in eight steps. J. Cell Biol..

[7] Campbell I.D., Humphries M.J. (2011). Integrin structure, activation, and interactions. Cold Spring Harb. Perspect. Biol..

[8] Rahmouni S., Lindner A., Rechenmacher F., Neubauer S., Sobahi T.R., Kessler H., Cavalcanti-Adam E.A., Spatz J.P. (2013). Hydrogel micropillars with integrin selective peptidomimetic functionalized nanopatterned tops: A new tool for the measurement of cell traction forces transmitted through alphavbeta3-or alpha5beta1-integrins. Adv. Mater..

[9] Dong X., Zhao B., Iacob R.E., Zhu J., Koksal A.C., Lu C., Engen J.R., Springer T.A. (2017). Force interacts with macromolecular structure in activation of TGF-beta. Nature.

[10] Hamidi H., Ivaska J. (2018). Every step of the way: Integrins in cancer progression and metastasis. Nat. Rev. Cancer.

[11] Takagi J., Petre B.M., Walz T., Springer T.A. (2002). Global conformational rearrangements in integrin extracellular domains in outside-in and inside-out signaling. Cell.

[12] Luo B.H., Carman C.V., Springer T.A. (2007). Structural basis of integrin regulation and signaling. Annu. Rev. Immunol..

[13] Humphries J.D., Byron A., Humphries M.J. (2006). Integrin ligands at a glance. J. Cell Sci..

[14] Munger J.S., Huang X., Kawakatsu H., Griffiths M.J., Dalton S.L., Wu J., Pittet J.F., Kaminski N., Garat C., Matthay M.A. (1999). The integrin alpha v beta 6 binds and activates latent TGF beta 1: A mechanism for regulating pulmonary inflammation and fibrosis. Cell.

[15] Shi M., Zhu J., Wang R., Chen X., Mi L., Walz T., Springer T.A. (2011). Latent TGF-beta structure and activation. Nature.

[16] Morikawa M., Derynck R., Miyazono K. (2016). TGF-beta and the TGF-beta Family: Context-Dependent Roles in Cell and Tissue Physiology. Cold Spring Harb. Perspect. Biol..

[17] Pickup M., Novitskiy S., Moses H.L. (2013). The roles of TGFβ in the tumour microenvironment. Nat. Rev. Cancer.

[18] Wu M.Y., Hill C.S. (2009). Tgf-beta superfamily signaling in embryonic development and homeostasis. Dev. Cell.

[19] Taipale J., Saharinen J., Keski-Oja J. (1998). Extracellular matrix-associated transforming growth factor-beta: Role in cancer cell growth and invasion. Adv. Cancer Res..

[20] Katz L.H., Li Y., Chen J.-S., Muñoz N.M., Majumdar A., Chen J., Mishra L. (2013). Targeting TGF-β signaling in cancer. Expert Opin. Ther. Targets.

[21] Derynck R., Zhang Y.E. (2003). Smad-dependent and Smad-independent pathways in TGF-beta family signalling. Nature.

[22] Robertson I.B., Rifkin D.B. (2016). Regulation of the Bioavailability of TGF-beta and TGF-beta-Related Proteins. Cold Spring Harb. Perspect. Biol..

[23] Wang R., Zhu J., Dong X., Shi M., Lu C., Springer T.A. (2012). GARP regulates the bioavailability and activation of TGFbeta. Mol. Biol. Cell.

[24] Lienart S., Merceron R., Vanderaa C., Lambert F., Colau D., Stockis J., van der Woning B., De Haard H., Saunders M., Coulie P.G. (2018). Structural basis of latent TGF-beta1 presentation and activation by GARP on human regulatory T cells. Science.

[25] Stockis J., Dedobbeleer O., Lucas S. (2017). Role of GARP in the activation of latent TGF-beta1. Mol. Biosyst..

[26] Hinck A.P., Mueller T.D., Springer T.A. (2016). Structural Biology and Evolution of the TGF-beta Family. Cold Spring Harb. Perspect. Biol..

[27] Rifkin D.B. (2005). Latent transforming growth factor-beta (TGF-beta) binding proteins: Orchestrators of TGF-beta availability. J. Biol. Chem..

[28] Jenkins G. (2008). The role of proteases in transforming growth factor-β activation. Int. J. Biochem. Cell Biol..

[29] Hayashi H., Sakai T. (2012). Biological Significance of Local TGF-β Activation in Liver Diseases. Front. Physiol..

[30] Sheppard D. (2015). Epithelial-mesenchymal interactions in fibrosis and repair. Transforming growth factor-beta activation by epithelial cells and fibroblasts. Ann. Am. Thorac. Soc..

[31] Wakefield L.M., Winokur T.S., Hollands R.S., Christopherson K., Levinson A.D., Sporn M.B. (1990). Recombinant latent transforming growth factor beta 1 has a longer plasma half-life in rats than active transforming growth factor beta 1, and a different tissue distribution. J. Clin. Investig..

[32] Hyytiainen M., Penttinen C., Keski-Oja J. (2004). Latent TGF-beta binding proteins: Extracellular matrix association and roles in TGF-beta activation. Crit. Rev. Clin. Lab. Sci..

[33] Huang X.Z., Wu J.F., Cass D., Erle D.J., Corry D., Young S.G., Farese R.V., Sheppard D. (1996). Inactivation of the integrin beta 6 subunit gene reveals a role of epithelial integrins in regulating inflammation in the lung and skin. J. Cell Biol..

[34] Kaminski N., Allard J.D., Pittet J.F., Zuo F., Griffiths M.J., Morris D., Huang X., Sheppard D., Heller R.A. (2000). Global analysis of gene expression in pulmonary fibrosis reveals distinct programs regulating lung inflammation and fibrosis. Proc. Natl. Acad. Sci. USA.

[35] Yang Z., Mu Z., Dabovic B., Jurukovski V., Yu D., Sung J., Xiong X., Munger J.S. (2007). Absence of integrin-mediated TGFbeta1 activation in vivo recapitulates the phenotype of TGFbeta1-null mice. J. Cell Biol..

[36] Mu D., Cambier S., Fjellbirkeland L., Baron J.L., Munger J.S., Kawakatsu H., Sheppard D., Broaddus V.C., Nishimura S.L. (2002). The integrin alpha(v)beta8 mediates epithelial homeostasis through MT1-MMP-dependent activation of TGF-beta1. J. Cell Biol..

[37] Aluwihare P., Mu Z., Zhao Z., Yu D., Weinreb P.H., Horan G.S., Violette S.M., Munger J.S. (2009). Mice that lack activity of alphavbeta6-and alphavbeta8-integrins reproduce the abnormalities of Tgfb1-and Tgfb3-null mice. J. Cell Sci..

[38] Wu B.X., Li A., Lei L., Kaneko S., Wallace C., Li X., Li Z. (2017). Glycoprotein A repetitions predominant (GARP) positively regulates transforming growth factor (TGF) beta3 and is essential for mouse palatogenesis. J. Biol. Chem..

[39] Yang L.T., Kaartinen V. (2007). Tgfb1 expressed in the Tgfb3 locus partially rescues the cleft palate phenotype of Tgfb3 null mutants. Dev. Biol..

[40] Worthington J.J., Klementowicz J.E., Travis M.A. (2011). TGFbeta: A sleeping giant awoken by integrins. Trends Biochem. Sci..

[41] Travis M.A., Reizis B., Melton A.C., Masteller E., Tang Q., Proctor J.M., Wang Y., Bernstein X., Huang X., Reichardt L.F. (2007). Loss of integrin alpha(v)beta8 on dendritic cells causes autoimmunity and colitis in mice. Nature.

[42] Miettinen P.J., Ebner R., Lopez A.R., Derynck R. (1994). TGF-beta induced transdifferentiation of mammary epithelial cells to mesenchymal cells: Involvement of type I receptors. J. Cell Biol..

[43] Munger J.S., Harpel J.G., Giancotti F.G., Rifkin D.B. (1998). Interactions between growth factors and integrins: Latent forms of transforming growth factor-beta are ligands for the integrin alphavbeta1. Mol. Biol. Cell.

[44] Ludbrook S.B., Barry S.T., Delves C.J., Horgan C.M.T. (2003). The integrin alphavbeta3 is a receptor for the latency-associated peptides of transforming growth factors beta1 and beta3. Biochem. J..

[45] Asano Y., Ihn H., Yamane K., Jinnin M., Tamaki K. (2006). Increased expression of integrin alphavbeta5 induces the myofibroblastic differentiation of dermal fibroblasts. Am. J. Pathol..

[46] Tatler A.L., John A.E., Jolly L., Habgood A., Porte J., Brightling C., Knox A.J., Pang L., Sheppard D., Huang X. (2011). Integrin alphavbeta5-mediated TGF-beta activation by airway smooth muscle cells in asthma. J. Immunol..

[47] Dong X., Hudson N.E., Lu C., Springer T.A. (2014). Structural determinants of integrin beta-subunit specificity for latent TGF-beta. Nat. Struct. Mol. Biol..

[48] Annes J.P., Rifkin D.B., Munger J.S. (2002). The integrin alphaVbeta6 binds and activates latent TGFbeta3. FEBS Lett..

[49] Annes J.P., Chen Y., Munger J.S., Rifkin D.B. (2004). Integrin alphaVbeta6-mediated activation of latent TGF-beta requires the latent TGF-beta binding protein-1. J. Cell Biol..

[50] Wipff P.-J., Hinz B. (2008). Integrins and the activation of latent transforming growth factor β1—An intimate relationship. Eur. J. Cell Biol..

[51] Fontana L., Chen Y., Prijatelj P., Sakai T., Fassler R., Sakai L.Y., Rifkin D.B. (2005). Fibronectin is required for integrin alphavbeta6-mediated activation of latent TGF-beta complexes containing LTBP-1. FASEB J..

[52] Buscemi L., Ramonet D., Klingberg F., Formey A., Smith-Clerc J., Meister J.J., Hinz B. (2011). The single-molecule mechanics of the latent TGF-beta1 complex. Curr. Biol..

[53] Wipff P.J., Rifkin D.B., Meister J.J., Hinz B. (2007). Myofibroblast contraction activates latent TGF-beta1 from the extracellular matrix. J. Cell Biol..

[54] Shull M.M., Ormsby I., Kier A.B., Pawlowski S., Diebold R.J., Yin M., Allen R., Sidman C., Proetzel G., Calvin D. (1992). Targeted disruption of the mouse transforming growth factor-β1 gene results in multifocal inflammatory disease. Nature.

[55] Kulkarni A.B., Huh C.G., Becker D., Geiser A., Lyght M., Flanders K.C., Roberts A.B., Sporn M.B., Ward J.M., Karlsson S. (1993). Transforming growth factor beta 1 null mutation in mice causes excessive inflammatory response and early death. Proc. Natl. Acad. Sci. USA.

[56] Li M.O., Wan Y.Y., Flavell R.A. (2007). T cell-produced transforming growth factor-beta1 controls T cell tolerance and regulates Th1-and Th17-cell differentiation. Immunity.

[57] Fahlen L., Read S., Gorelik L., Hurst S.D., Coffman R.L., Flavell R.A., Powrie F. (2005). T cells that cannot respond to TGF-beta escape control by CD4(+)CD25(+) regulatory T cells. J. Exp. Med..

[58] Nakamura K., Kitani A., Strober W. (2001). Cell contact-dependent immunosuppression by CD4(+)CD25(+) regulatory T cells is mediated by cell surface-bound transforming growth factor beta. J. Exp. Med..

[59] Tran D.Q., Andersson J., Wang R., Ramsey H., Unutmaz D., Shevach E.M. (2009). GARP (LRRC32) is essential for the surface expression of latent TGF-beta on platelets and activated FOXP3+ regulatory T cells. Proc. Natl. Acad. Sci. USA.

[60] Cuende J., Lienart S., Dedobbeleer O., van der Woning B., De Boeck G., Stockis J., Huygens C., Colau D., Somja J., Delvenne P. (2015). Monoclonal antibodies against GARP/TGF-beta1 complexes inhibit the immunosuppressive activity of human regulatory T cells in vivo. Sci. Transl. Med..

[61] Edwards J.P., Thornton A.M., Shevach E.M. (2014). Release of active TGF-β1 from the latent TGF-β1/GARP complex on T regulatory cells is mediated by integrin β8. J. Immunol..

[62] Worthington J.J., Kelly A., Smedley C., Bauche D., Campbell S., Marie J.C., Travis M.A. (2015). Integrin alphavbeta8-Mediated TGF-beta Activation by Effector Regulatory T Cells Is Essential for Suppression of T-Cell-Mediated Inflammation. Immunity.

[63] Stockis J., Liénart S., Colau D., Collignon A., Nishimura S.L., Sheppard D., Coulie P.G., Lucas S. (2017). Blocking immunosuppression by human Tregs in vivo with antibodies targeting integrin αVβ8. Proc. Natl. Acad. Sci. USA.

[64] Reszka A.A., Hayashi Y., Horwitz A.F. (1992). Identification of amino acid sequences in the integrin beta 1 cytoplasmic domain implicated in cytoskeletal association. J. Cell Biol..

[65] Moyle M., Napier M.A., McLean J.W. (1991). Cloning and expression of a divergent integrin subunit beta 8. J. Biol. Chem..

[66] Rachidi S., Metelli A., Riesenberg B., Wu B.X., Nelson M.H., Wallace C., Paulos C.M., Rubinstein M.P., Garrett-Mayer E., Hennig M. (2017). Platelets subvert T cell immunity against cancer via GARP-TGFbeta axis. Sci. Immunol..

[67] Cambier S., Mu D.Z., O’Connell D., Boylen K., Travis W., Liu W.H., Broaddus V.C., Nishimura S.L. (2000). A role for the integrin alphavbeta8 in the negative regulation of epithelial cell growth. Cancer Res..

[68] Kelly A., Gunaltay S., McEntee C.P., Shuttleworth E.E., Smedley C., Houston S.A., Fenton T.M., Levison S., Mann E.R., Travis M.A. (2018). Human monocytes and macrophages regulate immune tolerance via integrin αvβ8-mediated TGFβ activation. J. Exp. Med..

[69] Reynolds L.E., Wyder L., Lively J.C., Taverna D., Robinson S.D., Huang X., Sheppard D., Hynes R.O., Hodivala-Dilke K.M. (2002). Enhanced pathological angiogenesis in mice lacking beta3 integrin or beta3 and beta5 integrins. Nat. Med..

[70] Rolli M., Fransvea E., Pilch J., Saven A., Felding-Habermann B. (2003). Activated integrin alphavbeta3 cooperates with metalloproteinase MMP-9 in regulating migration of metastatic breast cancer cells. Proc. Natl. Acad. Sci. USA.

[71] Brooks P.C., Stromblad S., Sanders L.C., von Schalscha T.L., Aimes R.T., Stetler-Stevenson W.G., Quigley J.P., Cheresh D.A. (1996). Localization of matrix metalloproteinase MMP-2 to the surface of invasive cells by interaction with integrin alpha v beta 3. Cell.

[72] Yu Q., Stamenkovic I. (2000). Cell surface-localized matrix metalloproteinase-9 proteolytically activates TGF-beta and promotes tumor invasion and angiogenesis. Genes Dev..

[73] Henderson N.C., Arnold T.D., Katamura Y., Giacomini M.M., Rodriguez J.D., McCarty J.H., Pellicoro A., Raschperger E., Betsholtz C., Ruminski P.G. (2013). Targeting of alphav integrin identifies a core molecular pathway that regulates fibrosis in several organs. Nat. Med..

[74] Truong H.H., Xiong J., Ghotra V.P., Nirmala E., Haazen L., Le Devedec S.E., Balcioglu H.E., He S., Snaar-Jagalska B.E., Vreugdenhil E. (2014). Beta1 integrin inhibition elicits a prometastatic switch through the TGFbeta-miR-200-ZEB network in E-cadherin-positive triple-negative breast cancer. Sci. Signal..

[75] Shi Y., Massague J. (2003). Mechanisms of TGF-beta signaling from cell membrane to the nucleus. Cell.

[76] Attisano L., Wrana J.L. (1996). Signal transduction by members of the transforming growth factor-beta superfamily. Cytokine Growth Factor Rev..

[77] Massague J. (2008). TGFbeta in Cancer. Cell.

[78] Wrana J.L., Attisano L., Wieser R., Ventura F., Massagué J. (1994). Mechanism of activation of the TGF-β receptor. Nature.

[79] Moustakas A., Lin H.Y., Henis Y.I., Plamondon J., O’Connor-McCourt M.D., Lodish H.F. (1993). The transforming growth factor beta receptors types I, II, and III form hetero-oligomeric complexes in the presence of ligand. J. Biol. Chem..

[80] Chacko B.M., Qin B., Correia J.J., Lam S.S., de Caestecker M.P., Lin K. (2001). The L3 loop and C-terminal phosphorylation jointly define Smad protein trimerization. Nat. Struct. Biol..

[81] De Crescenzo G., Hinck C.S., Shu Z., Zuniga J., Yang J., Tang Y., Baardsnes J., Mendoza V., Sun L., Lopez-Casillas F. (2006). Three key residues underlie the differential affinity of the TGFbeta isoforms for the TGFbeta type II receptor. J. Mol. Biol..

[82] Qian S.W., Burmester J.K., Tsang M.L., Weatherbee J.A., Hinck A.P., Ohlsen D.J., Sporn M.B., Roberts A.B. (1996). Binding affinity of transforming growth factor-beta for its type II receptor is determined by the C-terminal region of the molecule. J. Biol. Chem..

[83] Inman G.J., Nicolas F.J., Hill C.S. (2002). Nucleocytoplasmic shuttling of Smads 2, 3, and 4 permits sensing of TGF-beta receptor activity. Mol. Cell.

[84] Moustakas A., Heldin C.H. (2005). Non-Smad TGF-beta signals. J. Cell Sci..

[85] Yue J., Mulder K.M. (2000). Activation of the mitogen-activated protein kinase pathway by transforming growth factor-beta. Methods Mol. Biol..

[86] Connolly E.C., Freimuth J., Akhurst R.J. (2012). Complexities of TGF-beta targeted cancer therapy. Int. J. Biol. Sci..

[87] Yang L., Pang Y., Moses H.L. (2010). TGF-beta and immune cells: An important regulatory axis in the tumor microenvironment and progression. Trends Immunol..

[88] Derynck R., Akhurst R.J. (2007). Differentiation plasticity regulated by TGF-beta family proteins in development and disease. Nat. Cell Biol..

[89] Flavell R.A., Sanjabi S., Wrzesinski S.H., Licona-Limon P. (2010). The polarization of immune cells in the tumour environment by TGFbeta. Nat. Rev. Immunol..

[90] Calon A., Lonardo E., Berenguer-Llergo A., Espinet E., Hernando-Momblona X., Iglesias M., Sevillano M., Palomo-Ponce S., Tauriello D.V., Byrom D. (2015). Stromal gene expression defines poor-prognosis subtypes in colorectal cancer. Nat. Genet..

[91] Levy L., Hill C.S. (2006). Alterations in components of the TGF-beta superfamily signaling pathways in human cancer. Cytokine Growth Factor Rev..

[92] Villanueva A., Garcia C., Paules A.B., Vicente M., Megias M., Reyes G., de Villalonga P., Agell N., Lluis F., Bachs O. (1998). Disruption of the antiproliferative TGF-beta signaling pathways in human pancreatic cancer cells. Oncogene.

[93] Grady W.M., Myeroff L.L., Swinler S.E., Rajput A., Thiagalingam S., Lutterbaugh J.D., Neumann A., Brattain M.G., Chang J., Kim S.J. (1999). Mutational inactivation of transforming growth factor beta receptor type II in microsatellite stable colon cancers. Cancer Res..

[94] Glick A.B., Weinberg W.C., Wu I.-H., Quan W., Yuspa S.H. (1996). Transforming growth factor β1 suppresses genomic instability independent of a G1 arrest, p53, and Rb. Cancer Res..

[95] Sun L., Wu G., Willson J.K., Zborowska E., Yang J., Rajkarunanayake I., Wang J., Gentry L.E., Wang X.F., Brattain M.G. (1994). Expression of transforming growth factor beta type II receptor leads to reduced malignancy in human breast cancer MCF-7 cells. J. Biol. Chem..

[96] Ludlow A., Yee K.O., Lipman R., Bronson R., Weinreb P., Huang X., Sheppard D., Lawler J. (2005). Characterization of integrin beta6 and thrombospondin-1 double-null mice. J. Cell. Mol. Med..

[97] Hezel A.F., Deshpande V., Zimmerman S.M., Contino G., Alagesan B., O’Dell M.R., Rivera L.B., Harper J., Lonning S., Brekken R.A. (2012). TGF-beta and alphavbeta6 integrin act in a common pathway to suppress pancreatic cancer progression. Cancer Res..

[98] Owens D.M., Romero M.R., Gardner C., Watt F.M. (2003). Suprabasal α6β4 integrin expression in epidermis results in enhanced tumourigenesis and disruption of TGFβ signalling. J. Cell Sci..

[99] Guasch G., Schober M., Pasolli H.A., Conn E.B., Polak L., Fuchs E. (2007). Loss of TGFbeta signaling destabilizes homeostasis and promotes squamous cell carcinomas in stratified epithelia. Cancer Cell.

[100] Brabletz T., Kalluri R., Nieto M.A., Weinberg R.A. (2018). EMT in cancer. Nat. Rev. Cancer.

[101] Thiery J.P., Acloque H., Huang R.Y., Nieto M.A. (2009). Epithelial-mesenchymal transitions in development and disease. Cell.

[102] Naber H.P., Drabsch Y., Snaar-Jagalska B.E., ten Dijke P., van Laar T. (2013). Snail and Slug, key regulators of TGF-beta-induced EMT, are sufficient for the induction of single-cell invasion. Biochem. Biophys. Res. Commun..

[103] Polyak K., Weinberg R.A. (2009). Transitions between epithelial and mesenchymal states: Acquisition of malignant and stem cell traits. Nat Rev Cancer.

[104] Huber M.A., Kraut N., Beug H. (2005). Molecular requirements for epithelial-mesenchymal transition during tumor progression. Curr. Opin. Cell Biol..

[105] Dumont N., Bakin A.V., Arteaga C.L. (2003). Autocrine transforming growth factor-beta signaling mediates Smad-independent motility in human cancer cells. J. Biol. Chem..

[106] Feldkoren B., Hutchinson R., Rapoport Y., Mahajan A., Margulis V. (2017). Integrin signaling potentiates transforming growth factor-beta 1 (TGF-beta1) dependent down-regulation of E-Cadherin expression—Important implications for epithelial to mesenchymal transition (EMT) in renal cell carcinoma. Exp. Cell Res..

[107] Bhowmick N.A., Zent R., Ghiassi M., McDonnell M., Moses H.L. (2001). Integrin β1 Signaling Is Necessary for Transforming Growth Factor-β Activation of p38MAPK and Epithelial Plasticity. J. Biol. Chem..

[108] Marsh D., Dickinson S., Neill G.W., Marshall J.F., Hart I.R., Thomas G.J. (2008). Alpha vbeta 6 Integrin promotes the invasion of morphoeic basal cell carcinoma through stromal modulation. Cancer Res..

[109] Margadant C., Sonnenberg A. (2010). Integrin-TGF-beta crosstalk in fibrosis, cancer and wound healing. EMBO Rep..

[110] Giampieri S., Manning C., Hooper S., Jones L., Hill C.S., Sahai E. (2009). Localized and reversible TGFbeta signalling switches breast cancer cells from cohesive to single cell motility. Nat. Cell Biol..

[111] Moore K.M., Thomas G.J., Duffy S.W., Warwick J., Gabe R., Chou P., Ellis I.O., Green A.R., Haider S., Brouilette K. (2014). Therapeutic targeting of integrin alphavbeta6 in breast cancer. J. Natl. Cancer Inst..

[112] Allen M.D., Thomas G.J., Clark S., Dawoud M.M., Vallath S., Payne S.J., Gomm J.J., Dreger S.A., Dickinson S., Edwards D.R. (2014). Altered microenvironment promotes progression of preinvasive breast cancer: Myoepithelial expression of alphavbeta6 integrin in DCIS identifies high-risk patients and predicts recurrence. Clin. Cancer Res..

[113] Bates R.C., Bellovin D.I., Brown C., Maynard E., Wu B., Kawakatsu H., Sheppard D., Oettgen P., Mercurio A.M. (2005). Transcriptional activation of integrin beta6 during the epithelial-mesenchymal transition defines a novel prognostic indicator of aggressive colon carcinoma. J. Clin. Investig..

[114] Martins V.L., Caley M.P., Moore K., Szentpetery Z., Marsh S.T., Murrell D.F., Kim M.H., Avari M., McGrath J.A., Cerio R. (2016). Suppression of TGFbeta and Angiogenesis by Type VII Collagen in Cutaneous SCC. J. Natl. Cancer Inst..

[115] Yin J.J., Selander K., Chirgwin J.M., Dallas M., Grubbs B.G., Wieser R., Massague J., Mundy G.R., Guise T.A. (1999). TGF-beta signaling blockade inhibits PTHrP secretion by breast cancer cells and bone metastases development. J. Clin. Investig..

[116] Fournier P.G., Juarez P., Jiang G., Clines G.A., Niewolna M., Kim H.S., Walton H.W., Peng X.H., Liu Y., Mohammad K.S. (2015). The TGF-beta Signaling Regulator PMEPA1 Suppresses Prostate Cancer Metastases to Bone. Cancer Cell.

[117] Chiechi A., Waning D.L., Stayrook K.R., Buijs J.T., Guise T.A., Mohammad K.S. (2013). Role of TGF-β in breast cancer bone metastases. Adv. Biosci. Biotechnol..

[118] Dutta A., Li J., Lu H., Akech J., Pratap J., Wang T., Zerlanko B.J., FitzGerald T.J., Jiang Z., Birbe R. (2014). Integrin alphavbeta6 promotes an osteolytic program in cancer cells by upregulating MMP2. Cancer Res..

[119] Dutta A., Li J., Fedele C., Sayeed A., Singh A., Violette S.M., Manes T.D., Languino L.R. (2015). αvβ6 integrin is required for TGFβ1-mediated matrix metalloproteinase2 expression. Biochem. J..

[120] Nishida N., Yano H., Nishida T., Kamura T., Kojiro M. (2006). Angiogenesis in cancer. Vasc. Health Risk Manag..

[121] Schniewind B., Groth S., Sebens Muerkoster S., Sipos B., Schafer H., Kalthoff H., Fandrich F., Ungefroren H. (2007). Dissecting the role of TGF-beta type I receptor/ALK5 in pancreatic ductal adenocarcinoma: Smad activation is crucial for both the tumor suppressive and prometastatic function. Oncogene.

[122] Dickson M.C., Martin J.S., Cousins F.M., Kulkarni A.B., Karlsson S., Akhurst R.J. (1995). Defective haematopoiesis and vasculogenesis in transforming growth factor-beta 1 knock out mice. Development.

[123] Liu Z., Kobayashi K., van Dinther M., van Heiningen S.H., Valdimarsdottir G., van Laar T., Scharpfenecker M., Lowik C.W., Goumans M.J., Ten Dijke P. (2009). VEGF and inhibitors of TGFbeta type-I receptor kinase synergistically promote blood-vessel formation by inducing alpha5-integrin expression. J. Cell Sci..

[124] Elayadi A.N., Samli K.N., Prudkin L., Liu Y.H., Bian A., Xie X.J., Wistuba I.I., Roth J.A., McGuire M.J., Brown K.C. (2007). A peptide selected by biopanning identifies the integrin alphavbeta6 as a prognostic biomarker for nonsmall cell lung cancer. Cancer Res..

[125] Weis S.M., Cheresh D.A. (2011). αV integrins in angiogenesis and cancer. Cold Spring Harb. Perspect. Med..

[126] Silva R., D’Amico G., Hodivala-Dilke K.M., Reynolds L.E. (2008). Integrins: The keys to unlocking angiogenesis. Arterioscler. Thromb. Vasc. Biol..

[127] Zhu J., Motejlek K., Wang D., Zang K., Schmidt A., Reichardt L.F. (2002). Beta8 integrins are required for vascular morphogenesis in mouse embryos. Development.

[128] Tchaicha J.H., Mobley A.K., Hossain M.G., Aldape K.D., McCarty J.H. (2010). A mosaic mouse model of astrocytoma identifies alphavbeta8 integrin as a negative regulator of tumor angiogenesis. Oncogene.

[129] Massague J. (1990). The Transforming Growth Factor-beta Family. Annu. Rev. Cell Biol..

[130] Cox T.R., Bird D., Baker A.M., Barker H.E., Ho M.W., Lang G., Erler J.T. (2013). LOX-mediated collagen crosslinking is responsible for fibrosis-enhanced metastasis. Cancer Res..

[131] Hayashida T., Wu M.H., Pierce A., Poncelet A.C., Varga J., Schnaper H.W. (2007). MAP-kinase activity necessary for TGFbeta1-stimulated mesangial cell type I collagen expression requires adhesion-dependent phosphorylation of FAK tyrosine 397. J. Cell Sci..

[132] Lamar J.M., Iyer V., DiPersio C.M. (2008). Integrin alpha3beta1 potentiates TGFbeta-mediated induction of MMP-9 in immortalized keratinocytes. J. Investig. Dermatol..

[133] Bacman D., Merkel S., Croner R., Papadopoulos T., Brueckl W., Dimmler A. (2007). TGF-beta receptor 2 downregulation in tumour-associated stroma worsens prognosis and high-grade tumours show more tumour-associated macrophages and lower TGF-beta1 expression in colon carcinoma: A retrospective study. BMC Cancer.

[134] Van Aarsen L.A., Leone D.R., Ho S., Dolinski B.M., McCoon P.E., LePage D.J., Kelly R., Heaney G., Rayhorn P., Reid C. (2008). Antibody-mediated blockade of integrin alpha v beta 6 inhibits tumor progression in vivo by a transforming growth factor-beta-regulated mechanism. Cancer Res..

[135] Eberlein C., Kendrew J., McDaid K., Alfred A., Kang J.S., Jacobs V.N., Ross S.J., Rooney C., Smith N.R., Rinkenberger J. (2012). A human monoclonal antibody 264RAD targeting αvβ6 integrin reduces tumour growth and metastasis, and modulates key biomarkers in vivo. Oncogene.

[136] Eberlein C., Rooney C., Ross S.J., Farren M., Weir H.M., Barry S.T. (2015). E-Cadherin and EpCAM expression by NSCLC tumour cells associate with normal fibroblast activation through a pathway initiated by integrin alphavbeta6 and maintained through TGFbeta signalling. Oncogene.

[137] Reader C.S., Vallath S., Steele C.W., Haider S., Brentnall A., Desai A., Moore K.M., Jamieson N.B., Chang D., Bailey P. (2019). The integrin αvβ6 drives pancreatic cancer through diverse mechanisms and represents an effective target for therapy. J. Pathol..

[138] Ozdemir B.C., Pentcheva-Hoang T., Carstens J.L., Zheng X., Wu C.C., Simpson T.R., Laklai H., Sugimoto H., Kahlert C., Novitskiy S.V. (2014). Depletion of carcinoma-associated fibroblasts and fibrosis induces immunosuppression and accelerates pancreas cancer with reduced survival. Cancer Cell.

[139] Rhim A.D., Oberstein P.E., Thomas D.H., Mirek E.T., Palermo C.F., Sastra S.A., Dekleva E.N., Saunders T., Becerra C.P., Tattersall I.W. (2014). Stromal elements act to restrain, rather than support, pancreatic ductal adenocarcinoma. Cancer Cell.

[140] Öhlund D., Handly-Santana A., Biffi G., Elyada E., Almeida A.S., Ponz-Sarvise M., Corbo V., Oni T.E., Hearn S.A., Lee E.J. (2017). Distinct populations of inflammatory fibroblasts and myofibroblasts in pancreatic cancer. J. Exp. Med..

[141] Neuzillet C., Tijeras-Raballand A., Ragulan C., Cros J., Patil Y., Martinet M., Erkan M., Kleeff J., Wilson J., Apte M. (2019). Inter-and intra-tumoural heterogeneity in cancer-associated fibroblasts of human pancreatic ductal adenocarcinoma. J. Pathol..

[142] Park E.J., Prajuabjinda O., Soe Z.Y., Darkwah S., Appiah M.G., Kawamoto E., Momose F., Shiku H., Shimaoka M. (2019). Exosomal regulation of lymphocyte homing to the gut. Blood Adv..

[143] Shimaoka M., Kawamoto E., Gaowa A., Okamoto T., Park E.J. (2019). Connexins and Integrins in Exosomes. Cancers.

[144] Singh A., Fedele C., Lu H., Nevalainen M.T., Keen J.H., Languino L.R. (2016). Exosome-mediated Transfer of αvβ3 Integrin from Tumorigenic to Nontumorigenic Cells Promotes a Migratory Phenotype. J. Mol. Cancer Res..

[145] Chen X., Song C.-H., Feng B.-S., Li T.-L., Li P., Zheng P.-Y., Chen X.-M., Xing Z., Yang P.-C. (2011). Intestinal epithelial cell-derived integrin αβ6 plays an important role in the induction of regulatory T cells and inhibits an antigen-specific Th2 response. J. Leukoc. Biol..

[146] Fedele C., Singh A., Zerlanko B.J., Iozzo R.V., Languino L.R. (2015). The alphavbeta6 integrin is transferred intercellularly via exosomes. J. Biol. Chem..

[147] Languino L.R., Singh A., Prisco M., Inman G.J., Luginbuhl A., Curry J.M., South A.P. (2016). Exosome-mediated transfer from the tumor microenvironment increases TGFβ signaling in squamous cell carcinoma. Am. J. Transl. Res..

[148] Li M.O., Flavell R.A. (2008). TGF-beta: A master of all T cell trades. Cell.

[149] Travis M.A., Sheppard D. (2014). TGF-beta activation and function in immunity. Annu. Rev. Immunol..

[150] Batlle E., Massague J. (2019). Transforming Growth Factor-beta Signaling in Immunity and Cancer. Immunity.

[151] Gong D., Shi W., Yi S.-j., Chen H., Groffen J., Heisterkamp N. (2012). TGFβ signaling plays a critical role in promoting alternative macrophage activation. BMC Immunol..

[152] Zhang M., He Y., Sun X., Li Q., Wang W., Zhao A., Di W. (2014). A high M1/M2 ratio of tumor-associated macrophages is associated with extended survival in ovarian cancer patients. J. Ovarian Res..

[153] Lu H., Bowler N., Harshyne L.A., Craig Hooper D., Krishn S.R., Kurtoglu S., Fedele C., Liu Q., Tang H.Y., Kossenkov A.V. (2018). Exosomal alphavbeta6 integrin is required for monocyte M2 polarization in prostate cancer. Matrix Biol..

[154] Fridlender Z.G., Sun J., Kim S., Kapoor V., Cheng G., Ling L., Worthen G.S., Albelda S.M. (2009). Polarization of tumor-associated neutrophil phenotype by TGF-beta: “N1” versus “N2” TAN. Cancer Cell.

[155] Sagiv J.Y., Michaeli J., Assi S., Mishalian I., Kisos H., Levy L., Damti P., Lumbroso D., Polyansky L., Sionov R.V. (2015). Phenotypic diversity and plasticity in circulating neutrophil subpopulations in cancer. Cell Rep..

[156] Marcoe J.P., Lim J.R., Schaubert K.L., Fodil-Cornu N., Matka M., McCubbrey A.L., Farr A.R., Vidal S.M., Laouar Y. (2012). TGF-beta is responsible for NK cell immaturity during ontogeny and increased susceptibility to infection during mouse infancy. Nat. Immunol..

[157] Ghiringhelli F., Menard C., Terme M., Flament C., Taieb J., Chaput N., Puig P.E., Novault S., Escudier B., Vivier E. (2005). CD4+CD25+ regulatory T cells inhibit natural killer cell functions in a transforming growth factor-beta-dependent manner. J. Exp. Med..

[158] Kim B.G., Li C., Qiao W., Mamura M., Kasprzak B., Anver M., Wolfraim L., Hong S., Mushinski E., Potter M. (2006). Smad4 signalling in T cells is required for suppression of gastrointestinal cancer. Nature.

[159] Trapani J.A. (2005). The dual adverse effects of TGF-beta secretion on tumor progression. Cancer Cell.

[160] Shang B., Liu Y., Jiang S.-j., Liu Y. (2015). Prognostic value of tumor-infiltrating FoxP3+ regulatory T cells in cancers: A systematic review and meta-analysis. Sci. Rep..

[161] Marie J.C., Liggitt D., Rudensky A.Y. (2006). Cellular mechanisms of fatal early-onset autoimmunity in mice with the T cell-specific targeting of transforming growth factor-beta receptor. Immunity.

[162] Tone Y., Furuuchi K., Kojima Y., Tykocinski M.L., Greene M.I., Tone M. (2008). Smad3 and NFAT cooperate to induce Foxp3 expression through its enhancer. Nat. Immunol..

[163] Yang P., Li Q.J., Feng Y., Zhang Y., Markowitz G.J., Ning S., Deng Y., Zhao J., Jiang S., Yuan Y. (2012). TGF-beta-miR-34a-CCL22 signaling-induced Treg cell recruitment promotes venous metastases of HBV-positive hepatocellular carcinoma. Cancer Cell.

[164] Melton A.C., Bailey-Bucktrout S.L., Travis M.A., Fife B.T., Bluestone J.A., Sheppard D. (2010). Expression of alphavbeta8 integrin on dendritic cells regulates Th17 cell development and experimental autoimmune encephalomyelitis in mice. J. Clin. Investig..

[165] Gorelik L., Flavell R.A. (2001). Immune-mediated eradication of tumors through the blockade of transforming growth factor-beta signaling in T cells. Nat. Med..

[166] Tauriello D.V.F., Palomo-Ponce S., Stork D., Berenguer-Llergo A., Badia-Ramentol J., Iglesias M., Sevillano M., Ibiza S., Canellas A., Hernando-Momblona X. (2018). TGFbeta drives immune evasion in genetically reconstituted colon cancer metastasis. Nature.

[167] Budhu S., Schaer D.A., Li Y., Toledo-Crow R., Panageas K., Yang X., Zhong H., Houghton A.N., Silverstein S.C., Merghoub T. (2017). Blockade of surface-bound TGF-β on regulatory T cells abrogates suppression of effector T cell function in the tumor microenvironment. Sci. Signal..

[168] Herbst R.S., Soria J.C., Kowanetz M., Fine G.D., Hamid O., Gordon M.S., Sosman J.A., McDermott D.F., Powderly J.D., Gettinger S.N. (2014). Predictive correlates of response to the anti-PD-L1 antibody MPDL3280A in cancer patients. Nature.

[169] Hegde P.S., Karanikas V., Evers S. (2016). The Where, the When, and the How of Immune Monitoring for Cancer Immunotherapies in the Era of Checkpoint Inhibition. Clin. Cancer Res..

[170] Mariathasan S., Turley S.J., Nickles D., Castiglioni A., Yuen K., Wang Y., Kadel E.E., Koeppen H., Astarita J.L., Cubas R. (2018). TGFbeta attenuates tumour response to PD-L1 blockade by contributing to exclusion of T cells. Nature.

[171] Neuzillet C., Tijeras-Raballand A., Cohen R., Cros J., Faivre S., Raymond E., de Gramont A. (2015). Targeting the TGFbeta pathway for cancer therapy. Pharmacol. Ther..

[172] Herbertz S., Sawyer J.S., Stauber A.J., Gueorguieva I., Driscoll K.E., Estrem S.T., Cleverly A.L., Desaiah D., Guba S.C., Benhadji K.A. (2015). Clinical development of galunisertib (LY2157299 monohydrate), a small molecule inhibitor of transforming growth factor-beta signaling pathway. Drug Des. Dev. Ther..

[173] Hazelbag S., Kenter G.G., Gorter A., Dreef E.J., Koopman L.A., Violette S.M., Weinreb P.H., Fleuren G.J. (2007). Overexpression of the alpha v beta 6 integrin in cervical squamous cell carcinoma is a prognostic factor for decreased survival. J. Pathol..

[174] Sipos B., Hahn D., Carceller A., Piulats J., Hedderich J., Kalthoff H., Goodman S.L., Kosmahl M., Klöppel G. (2004). Immunohistochemical screening for β6-integrin subunit expression in adenocarcinomas using a novel monoclonal antibody reveals strong up-regulation in pancreatic ductal adenocarcinomas in vivo and in vitro. Histopathology.

[175] Posey J.A., Khazaeli M.B., DelGrosso A., Saleh M.N., Lin C.Y., Huse W., LoBuglio A.F. (2001). A pilot trial of Vitaxin, a humanized anti-vitronectin receptor (anti alpha v beta 3) antibody in patients with metastatic cancer. Cancer Biother. Radiopharm..

[176] Hersey P., Sosman J., O’Day S., Richards J., Bedikian A., Gonzalez R., Sharfman W., Weber R., Logan T., Buzoianu M. (2010). A randomized phase 2 study of etaracizumab, a monoclonal antibody against integrin alpha(v)beta(3), + or − dacarbazine in patients with stage IV metastatic melanoma. Cancer.

[177] Elez E., Kocakova I., Hohler T., Martens U.M., Bokemeyer C., Van Cutsem E., Melichar B., Smakal M., Csoszi T., Topuzov E. (2015). Abituzumab combined with cetuximab plus irinotecan versus cetuximab plus irinotecan alone for patients with KRAS wild-type metastatic colorectal cancer: The randomised phase I/II POSEIDON trial. Ann. Oncol..

[178] Stupp R., Hegi M.E., Gorlia T., Erridge S.C., Perry J., Hong Y.K., Aldape K.D., Lhermitte B., Pietsch T., Grujicic D. (2014). Cilengitide combined with standard treatment for patients with newly diagnosed glioblastoma with methylated MGMT promoter (CENTRIC EORTC 26071-22072 study): A multicentre, randomised, open-label, phase 3 trial. Lancet Oncol..

[179] Nabors L.B., Fink K.L., Mikkelsen T., Grujicic D., Tarnawski R., Nam D.H., Mazurkiewicz M., Salacz M., Ashby L., Zagonel V. (2015). Two cilengitide regimens in combination with standard treatment for patients with newly diagnosed glioblastoma and unmethylated MGMT gene promoter: Results of the open-label, controlled, randomized phase II CORE study. Neuro-Oncology.

[180] Weller M., Nabors L.B., Gorlia T., Leske H., Rushing E., Bady P., Hicking C., Perry J., Hong Y.-K., Roth P. (2016). Cilengitide in newly diagnosed glioblastoma: Biomarker expression and outcome. Oncotarget.

[181] Hargadon K.M., Johnson C.E., Williams C.J. (2018). Immune checkpoint blockade therapy for cancer: An overview of FDA-approved immune checkpoint inhibitors. Int. Immunopharmacol..

[182] Bellmunt J., de Wit R., Vaughn D.J., Fradet Y., Lee J.L., Fong L., Vogelzang N.J., Climent M.A., Petrylak D.P., Choueiri T.K. (2017). Pembrolizumab as Second-Line Therapy for Advanced Urothelial Carcinoma. N. Engl. J. Med..

[183] Rosenberg J.E., Hoffman-Censits J., Powles T., van der Heijden M.S., Balar A.V., Necchi A., Dawson N., O’Donnell P.H., Balmanoukian A., Loriot Y. (2016). Atezolizumab in patients with locally advanced and metastatic urothelial carcinoma who have progressed following treatment with platinum-based chemotherapy: A single-arm, multicentre, phase 2 trial. Lancet.

[184] Balar A.V., Galsky M.D., Rosenberg J.E., Powles T., Petrylak D.P., Bellmunt J., Loriot Y., Necchi A., Hoffman-Censits J., Perez-Gracia J.L. (2017). Atezolizumab as first-line treatment in cisplatin-ineligible patients with locally advanced and metastatic urothelial carcinoma: A single-arm, multicentre, phase 2 trial. Lancet.

[185] Michot J.M., Bigenwald C., Champiat S., Collins M., Carbonnel F., Postel-Vinay S., Berdelou A., Varga A., Bahleda R., Hollebecque A. (2016). Immune-related adverse events with immune checkpoint blockade: A comprehensive review. Eur. J. Cancer.

[186] Bertrand A., Kostine M., Barnetche T., Truchetet M.E., Schaeverbeke T. (2015). Immune related adverse events associated with anti-CTLA-4 antibodies: Systematic review and meta-analysis. BMC Med..

